# Beta Adrenergic Regulation of Intrapulmonary Arteriovenous Anastomoses in Intact Rat and Isolated Rat Lungs

**DOI:** 10.3389/fphys.2017.00218

**Published:** 2017-04-19

**Authors:** Melissa L. Bates, Joseph E. Jacobson, Marlowe W. Eldridge

**Affiliations:** ^1^Critical Care Division and the John Rankin Laboratory of Pulmonary Medicine, Department of Pediatrics, University of IowaIowa City, IA, USA; ^2^Department of Health and Human Physiology, University of IowaIowa City, IA, USA; ^3^Michigan State University College of Human MedicineEast Lansing, MI, USA; ^4^Departments of Biomedical Engineering and Kinesiology, University of WisconsinMadison, WI, USA

**Keywords:** arteriovenous shunt, pulmonary blood flow, hypoxia

## Abstract

Intrapulmonary arteriovenous anastomoses (IPAVA) allow large diameter particles of venous origin to bypass the pulmonary capillary bed and embolize the systemic arterial circulation. IPAVA have been routinely observed in healthy humans with exercise, hypoxia, and catecholamine infusion, but the mechanism by which they are recruited is not well-defined. We hypothesized that beta-adrenergic receptor stimulation recruits IPAVA and that receptor blockade would limit hypoxia-induced IPAVA recruitment. To test our hypothesis, we evaluated the transpulmonary passage of microspheres in intact rats and isolated rats lung infused with the beta-adrenergic receptor agonist isoproterenol. We also evaluated IPAVA recruitment in intact rats with hypoxia and the beta-adrenergic receptor blocker propranolol. We found that IPAVA are recruited in the intact rat by isoproterenol and their recruitment by hypoxia can be minimized by propranolol, suggesting a role for the adrenergic system in the recruitment of IPAVA by hypoxia. IPAVA recruitment is completely abolished by ventilation with 100% oxygen. Isoproterenol also recruits IPAVA in isolated rat lungs. The fact that isoproterenol can recruit IPAVA in isolated lungs, without increased pulmonary flow, suggests that elevated cardiac output is not required for IPAVA recruitment.

## Introduction

Intrapulmonary arteriovenous anastomoses (IPAVA) directly connect arteries and veins in the lung, thereby bypassing the pulmonary capillary bed (Tobin and Zariquiey, 1950; Tobin, 1966; Lovering et al., 2007). These pathways, which are ≥70 μm in diameter in the rat (Bates et al., 2012), and are recruited by exercise (Eldridge et al., 2004; Lovering et al., 2006, 2008a,b, 2009; Bates et al., 2014a; Cameron Norris et al., 2014), hypoxia (Lovering et al., 2006; Bates et al., 2012, 2014a), and exogenous catecholamines (Bryan et al., 2012; Laurie et al., 2012; Elliott et al., 2014) in humans. They provide a potential route for thrombi and other particles to bypass the lung filter and embolize the systemic vasculature (Lovering et al., 2010; Laurie et al., 2011). Although the physiological conditions that lead to IPAVA recruitment are well-described, the mechanism by which they are recruited is unclear.

Saline contrast echocardiography has been used to evaluate the relationship between pulmonary hemodynamics and IPAVA recruitment. Briefly, this technique measures the transpulmonary passage of saline microbubbles to detect patent IPAVA. In the normoxic, resting lung, venous-injected microbubbles lodge in the lung vasculature and do not return to the left heart. When IPAVA are perfused, microbubbles transit the lung, and can be observed in the left ventricle. Elliot et al. observed that epinephrine infusion recruits IPAVA and increases physiological shunt (Elliott et al., 2014). This group also observed a relationship between the dose of epinephrine and the intensity of the transpulmonary passage of agitated saline microbubbles, concluding that there is a dose-response relationship between cardiac output and IPAVA recruitment (Laurie et al., 2012). Similarly, Bryan et al. demonstrated a relationship between the intensity of right-to-left bubble passage and cardiac output with dobutamine infusion (Bryan et al., 2012). These authors suggest that IPAVA are mechanically recruited by increased cardiac output.

However, an alternative explanation for the results is that the increase in cardiac output is only coincidental with IPAVA recruitment and that the direct biochemical effects of epinephrine and dobutamine mediate the opening of IPAVA. In support of this, the transpulmonary passage of microbubbles during high intensity exercise can be prevented or reversed by hyperoxia despite the fact that the cardiac output remains elevated (Lovering et al., 2008b). Furthermore, we recently quantified IPAVA recruitment in humans breathing hypoxic gas at rest and during exercise using ^99m^Tc-labeled albumin particles (Bates et al., 2014a). We found that the addition of exercise, which increases cardiac output, does not increase IPAVA recruitment in hypoxia. Instead, blood flow through these pathways is higher with resting, hypoxic breathing compared to exercise. This suggests that elevated cardiac output is not always associated with increased blood flow through IPAVA. The differences between studies using saline contrast echocardiography and solid ^99m^Tc-MAA may be related to the different methodologies used. The association between microbubble passage and cardiac output may be explained by the stability of the agitated saline microbubbles. Hackett et al. demonstrated that the stability of saline microbubbles is dependent on the transit time (Hackett et al., 2016), such that more bubbles may survive transit across the lung when flow is higher. This may lead to the interpretation that there is little or no IPAVA perfusion when the transit time is longer and few bubbles survive to be detected in the left ventricle. This highlights the need to evaluate mechanisms of IPAVA recruitment with solid particle techniques, which are highly stable and not subject to limitations related to their lifespan.

Both dobutamine and epinephrine are β-receptor agonists and cause vasodilation. We, therefore, hypothesized that activation of β-adrenergic receptors is responsible for the opening or dilation of IPAVA and that an increase in cardiac output is not necessary for IPAVA recruitment. We also hypothesized that IPAVA opening would be reduced in hypoxia by β-receptor blockade. To test our hypothesis, we measured the transpulmonary passage of microspheres in isolated, perfused rat lungs, taking care to match pulmonary artery pressure and flow between groups. Right-to-left microsphere passage occurred only in isoproterenol perfused lungs, but not in lungs with elevated pulmonary artery pressure and flow. We also measured the transpulmonary passage of large diameter, venous-injected microspheres in anesthetized, room air ventilated rats infused with either saline or isoproterenol and found substantial microsphere passage with β-receptor stimulation. Consistent with other observations of IPAVA with exercise and catecholamine infusion, 100% O_2_ eliminated blood flow through these pathways. β-receptor blockade with propranolol also reduced microsphere passage in hypoxic rats. Taken together, these studies demonstrate that β–agonists recruit IPAVA independent of elevated pulmonary artery pressure and flow and that β–blockade substantially attenuates hypoxia-induced IPAVA opening.

## Methods

Adult male Sprague Dawley rats (200–450 g) were obtained for study (Harlan, Inc.) and maintained on a typical 12-h light:dark cycle with a standard chow diet and water offered *ad libitum*. At the time of study, all rats were anesthetized with ketamine (50–100 mg/kg) and xylazine (10 mg/kg). The University of Wisconsin-Madison School of Medicine and Public Health's Institutional Animal Care and Use Committee approved this protocol.

### Experiment 1: β-agonist induced IPAVA recruitment in isolated lungs

Our isolated lung preparation has been well-described in detail previously (Lovering et al., 2007; Stickland et al., 2007; Bates et al., 2012). Briefly, rats (*n* = 6–9, each group) were secured supine, the trachea was cannulated, the femoral vein was catheterized, and 6,000 U/kg heparin was injected intravenously. To prevent clot formation during lung isolation, blood was flushed from the rat by cutting the femoral artery and simultaneously perfusing the femoral vein with 10 mL of isotonic saline. The lungs were then inflated with 5 mL of air and the chest wall was removed. A fluid filled catheter was placed in the pulmonary artery and connected to a continuous flow reservoir, allowing the fluid column height to remain constant and the lungs to be gravitationally perfused. In this way, the pulmonary artery perfusion pressure is determined by the height of the fluid column. An additional fluid filled catheter was placed in the left atrium for the collection of the lung effluent and maintained at the height of the left atrium so that the left outlet pressure was 0 cmH_2_O. The lungs were mechanically ventilated 30–45 times/min to a peak inflation pressure of 15 cmH_2_O with 5 cmH_2_O end expiratory pressure.

#### Experimental design

Isolated lungs were perfused for 20 min under the following conditions:

1. 3% hetastarch, 20 cmH_2_O perfusion pressure
2. 3% hetastarch, 40 cmH_2_O perfusion pressure
3. 100 μM isoproterenol in 3% hetastarch, 20 cmH_2_O perfusion pressure
4. 100 μM isoproterenol and 100 μM propranolol in 3% hetastarch, 20 cmH_2_O perfusion pressure.

Rats in condition 4 were also pre-treated with propranolol 20 min before lung isolation (0.8 mg/kg, i.p.). Following 20 min of perfusion, total lung perfusate flow was quantified by collecting the left atrial effluent in a graduated cylinder for 2 min. Consistent with our previous studies in isolated lungs (Lovering et al., 2007; Stickland et al., 2007; Bates et al., 2012), a single bolus of 1 × 10^5^ 50 μm green fluorescent microspheres was then injected into the pulmonary artery catheter and the entire left atrial effluent was collected.

#### Quantification of IPAVA recruitment

The entire left atrial effluent was vortexed, and vacuum filtered through a filter with 8 μm pores. The filters were then imaged with a fluorescent microscope and the number of microspheres was counted and expressed as a percent of the number injected.

### Experiment 2: β-agonist induced IPAVA recruitment in intact rats

#### Surgical preparation

Rats were secured supine on a homeothermic blanket to maintain body temperature (37–38°C), intubated, mechanically ventilated with 21% O_2_ (8–12 mL/kg), and hyperventilated at a rate sufficient to abolish spontaneous ventilation. The neck was exposed and a custom Tygon catheter (0.010 in. ID, 0.030 in. OD), connected to a pressure transducer (Hospira, Lake Forest, IL), was introduced into the right ventricle via the jugular vein for the injection of microspheres and measurement of right ventricular pressure. A second polyethylene catheter (Sai Technologies, PE50) was introduced into the superior vena cava via the same jugular vein for the infusion of drugs. A final polyethylene catheter (Sai Technologies, PE10) was introduced via the carotid artery into the left ventricle for blood sampling, blood pressure monitoring, and systemic microsphere injection. At the end of the experiment, rats were euthanized by exsanguination under anesthesia, secondary to the removal of the liver and heart. As we have done previously, each heart was examined for the presence of a patent foramen ovale (PFO; Bates et al., 2012), which could allow right-to-left passage of microspheres independent of IPAVA recruitment. Two rats with a PFO were excluded from analysis.

#### Experimental design

Groups of rats (*n* = 6, each group) were assigned to the following combinations of gas exposure and drug treatment:

1. 21% O_2_ and 0.9% NaCl (i.v.)
2. 21% O_2_ and Isoproterenol (1 μg/kg/min, i.v.)
3. 21% O_2_ and Isoproterenol (1 μg/kg/min, i.v.) + Propranolol (0.8 mg/kg, i.p.)
4. 100% O_2_ and Isoproterenol (1 μg/kg/min, i.v.)
5. 10% O_2_ and 0.9% NaCl (i.v.)
6. 10% O_2_ and Propranolol (0.8 mg/kg, i.p.).

Saline and isoproterenol were infused at a rate of 100 μL/min and propranolol was given 20 min prior to beginning the gas exposure. After 10 min of gas exposure and drug infusion, blood was drawn from the left ventricle into a heparinized syringe for blood gas analysis. Boluses of 1 × 10^6^ red and green fluorescent 15 μm (±2%) microspheres (Duke Scientific) were injected into the left and right ventricles, respectively. It is important to note that smaller, 15 μm microspheres were used here because they do not traverse the lungs in normoxic rats or isolated lungs, and also do not impact cardiac output and right ventricular systolic pressure (Bates et al., 2012). Rats were then euthanized and organs were removed for analysis.

In a second group of rats (*n* = 3, each group) exposed to conditions 1, 2, 4, and 5 for 10 min, we confirmed that the large diameter microspheres we used in our previous hypoxia studies would also traverse the lungs of animals infused with isoproterenol (Bates et al., 2012). After 10 min of exposure to the experimental conditions, a bolus of 1 × 10^6^ green and red fluorescent microspheres (2 × 10^5^ each of 10 ± 18, 15 ± 3, 25 ± 12, 50 ± 12, and 70 ± 7% μm microspheres) was injected into the right ventricle. Again, because these larger microspheres can impact hemodynamics, they were used to determine whether IPAVA of these sizes were recruited but not to quantify IPAVA flow. After microsphere injections, we inspected the syringes and catheters to verify that they did not retain fluorescent particles.

#### Quantification of IPAVA recruitment

For experiments using a single size of microsphere, the liver was hydrolyzed in 5N NaOH for 24 h at 37–40°C and then vortexed and vacuum filtered through a filter with 8 μm pores (Millipore, Billerica, MA). Blank samples were filtered at random between liver samples to verify that we did not transfer microspheres between samples. Microspheres trapped in the filters were imaged using a fluorescent microscope and counted. Considering the number of microspheres injected in the right (RV) and left ventricle (LV) that were found trapped in the liver, the percent of 15 μm microspheres traversing the lung was quantified accordingly (Stickland et al., 2007; Bates et al., 2012):

$$\begin{array}{lll} \text{IPAVA Perfusion }\left(\%\right) & = & \;\frac{\#\text{ Spheres injected in LV}}{\#\text{ LV spheres found in liver}}\\ \text{ x }\frac{\#\text{ RV spheres found in liver}}{\#\text{ spheres injected in RV}} \end{array}$$

For samples containing microspheres of multiple diameters, the number of microspheres was counted and the diameter of each microsphere was measured (ImageJ), but the fraction of microspheres traversing the lung was not quantified. A subset of filters was also manually imaged. The microspheres trapped on these filters were manually counted by two investigators (MLB and JEJ) was found to be no more than 2 microspheres different from the number quantified with ImageJ. The liver was used as the site of microsphere capture because it receives ~20–25% of the cardiac output (Johnston and Owen, 1977). With 1 × 10^6^ microspheres injected into the mixed venous circulation, a difference in IPAVA flow of 1% would yield a difference in 2,500 liver-trapped particles.

#### Statistical analysis

All data are displayed as mean ± standard deviation unless otherwise noted. Between group differences were evaluated by analysis of variance, with a *post-hoc* Dunnett test to compare each group's response to the control (Minitab, State College, PA). Significance was determined when *p* < 0.05.

## Results

### Isolated lung studies

**Figure 1:**
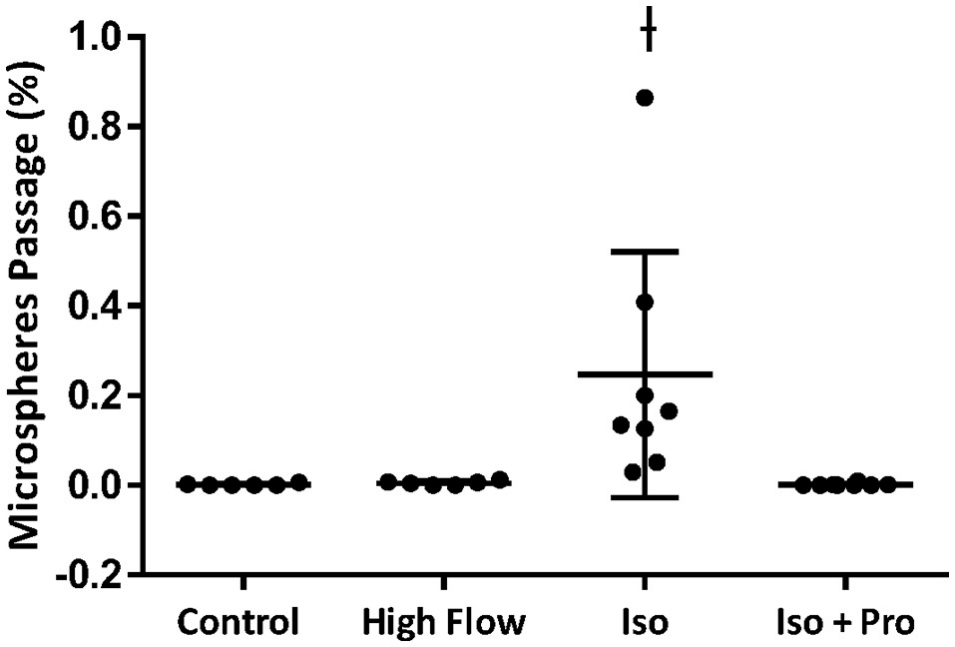
**Percent transpulmonary passage of venous-injected microspheres in the isolated rat lung**. Microsphere passage was trivial in control lungs (20 cmH_2_O pressure, 3.3 ± 1.1 mL/min) and lungs perfused with higher flow (40 cmH_2_O pressure, 6.9 ± 1.0 mL/min). Addition of 100 μM isoproterenol (Iso) to the perfusate increased the transpulmonary passage of 50 μm microspheres. This was eliminated by the addition of the beta blocker propranolol (Iso + Pro). ^†^Indicates *p* < 0.05 compared to control.

Perfusion with 3% hetastarch at 40 cmH_2_O pulmonary artery pressure increased the rate of perfusate flow across the lung compared to 20 cmH_2_O (6.9 ± 1.0 vs. 3.3 ± 1.1 mL/min, *p* < 0.001). Flow across lungs perfused with isoproterenol was similar to control lungs (4.3 ± 1.1 mL/min, *p* > 0.05). The injection of 50 μm microspheres decreased perfusate flow 29.7 ± 25.1%. Congruent with our previous observations (Bates et al., 2012), the percent passage of microspheres across the lung with 20 cmH_2_O perfusion pressure was trivial (0.001 ± 0.002%) and this was not increased by raising the perfusion pressure (0.005 ± 0.005%, *p* > 0.05; Figure 1). Perfusion with isoproterenol at 20 cmH_2_O increased microsphere passage compared to the hetastarch control (0.221 ± 0.269%, *p* < 0.05) and this was prevented with pre-treatment with propranolol (0.001 ± 0.003%, *p* > 0.05).

### Intact rat studies

**Figure 2:**
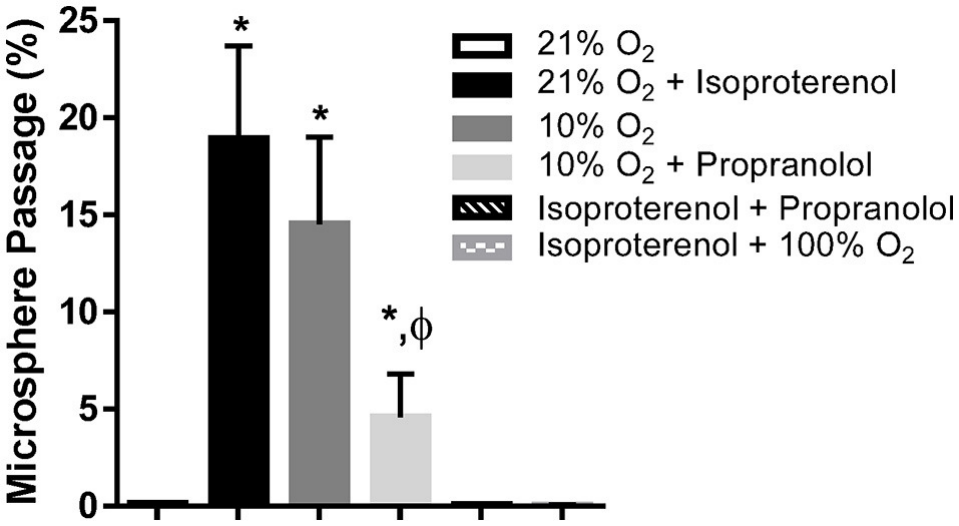
**Percent transpulmonary passage of venous-injected microspheres in the intact rat**. Data are presented as ordered in the legend. Hypoxia (10% O_2_) and isoproterenol infusion recruited intrapulmonary arteriovenous anastomoses (IPAVA), evidenced by an increase in the percent of venous-injected microspheres traversing the lungs and embolizing the liver. Propranolol pre-treatment minimized hypoxia- and isoproterenol-induced IPAVA recruitment. 100% O_2_ eliminated isoproterenol-induced IPAVA perfusion. ^*^Indicates *p* < 0.05 compared to 21% O_2_ and ϕ indicates *p* < 0.05 compared to 10% O_2_.

Hemodynamic parameters and blood gas values for our intact rat studies are given in Table 1. As expected, hypoxia depressed PaO_2_ and hyperoxia raised PaO_2_, compared to 21% O_2_. Infusion with isoproterenol increased heart rate. As we demonstrated previously, hypoxic ventilation increased transpulmonary passage of 15 μm microspheres (14.5 ± 4.5%), compared to room air (0.11 ± 0.03%, *p* < 0.001; Figure 2). Similarly, isoproterenol increased microsphere passage in normoxic rats (18.9 ± 4.8%, *p* < 0.001). Isoproterenol-induced IPAVA recruitment was eliminated by ventilation with 100% O_2_ and pre-treatmentwith propranolol (0.03 ± 0.04 and 0.07 ± 0.04%, *p* > 0.05 compared to room air control). Pre-treatment with propranolol also significantly reduced hypoxia-induced IPAVA perfusion (4.6 ± 2.2%, *p* < 0.05).

**Table 1:** **Hemodynamic and blood gas parameters for intact rat experiments**.

	**Heart rate (bpm)**	**LV systolic pressure (mmHg)**	**RV systolic pressure (mmHg)**	**PaO_2_ (mmHg)**	**PaCO_2_(mmHg)**	**pH**
21% O_2_	303 ± 83	84 ± 16	22 ± 4	92 ± 25	29 ± 8	7.39 ± 0.09
10% O_2_	282 ± 61	60 ± 14	24 ± 10	41 ± 6^**^	23 ± 7	7.40 ± 0.14
100% O_2_	296 ± 21	91 ± 20	15 ± 3	313 ± 62^**^	23 ± 2	7.41 ± 0.07
21% O_2_ + Isoproterenol	467 ± 76^**^	75 ± 14	23 ± 3	74 ± 15	25 ± 9	7.37 ± 0.16
21% O_2_ + Isoproterenol + Propranolol	409 ± 97	68 ± 22	30 ± 2	83 ± 12	31 ± 3	7.41 ± 0.14
100% O_2_ + Isoproterenol	444 ± 55^**^	69 ± 18	19 ± 4	160 ± 61^**^	25 ± 5	7.42 ± 0.03
10% O_2_ + Propranolol	356 ± 41	78 ± 14	22 ± 6	41 ± 9^**^	27 ± 5	7.39 ± 0.20

** *Indicates values that are >21% O_2_ (p < 0.05). bpm, beats per m; LV, left ventricle; RV, right ventricle; PaO_2_, arterial partial pressure of oxygen; PaCO_2_, arterial partial pressure of carbon dioxide*.

In experiments with multiple size microspheres, a trivial number of microspheres traversed the lung with normoxic and hyperoxic ventilation, and only the smallest microspheres were found in the liver (Table 2). Hypoxic ventilation and isoproterenol infusion increased the passage of all microsphere sizes, including 70 μm microspheres. The passage of larger microspheres with isoproterenol infusion and hypoxic ventilation was eliminated by hyperoxia and propranolol, respectively.

**Table 2:** **Number of venous-injected microspheres found in the liver of intact rats**.

**Condition**	**10 μm**	**15 μm**	**25 μm**	**50 μm**	**70 μm**
21% O_2_	30 ± 10	11 ± 6	10 ± 11	0 ± 0	0 ± 0
10% O_2_	1, 073±836^**^	698 ± 709^**^	235 ± 259^**^	379 ± 910^**^	22 ± 14^**^
100% O_2_	16 ± 2	5 ± 4	0 ± 0	0 ± 0	0 ± 0
21% O_2_ + Isoproterenol	2, 748 ± 4, 505^**^	1, 924 ± 2, 602^**^	1, 179 ± 829^**^	307 ± 147^**^	49 ± 38^**^
100% O_2_ + Isoproterenol	20 ± 15	6 ± 5	6 ± 9	0 ± 0	0 ± 0
10% O_2_ + Propranolol	13 ± 7	5 ± 4	9 ± 14	0 ± 0	0 ± 0

** *Indicates values that are >21% O_2_ (p < 0.05)*.

## Discussion

Several recent studies have investigated the relative importance of cardiac output on IPAVA recruitment in humans. These studies have been absolutely critical in demonstrating the external stimuli that recruit IPAVA, but they are mechanistically complicated in that many of these stimuli have impacts on both vascular tone and hemodynamics. Therefore, in order to separate the effects on vascular tone and cardiac output, we used both intact animal and isolated lung approaches. Here, we demonstrate that the β-adrenergic agonist isoproterenol recruits IPAVA both in the intact animal and isolated lung, where pulmonary artery flow and pressure can be controlled.

We previously used a similar approach to investigate the ability of alveolar hypoxia to recruit IPAVA in intact rats and isolated lungs (Bates et al., 2012). In these earlier studies, hypoxia caused IPAVA recruitment in the intact rat, allowing the transpulmonary passage of large diameter microspheres, but failed to increase microsphere passage in the isolated lung. We concluded that the signal for IPAVA recruitment might be neural or humoral and, therefore, IPAVA could not be recruited when the lung was removed from the body. Indeed, although the transpulmonary passage of 25–50 μm microspheres has been noted previously in human, dog, baboon, and rat lungs, the fraction is trivial. This motivated us to consider that, unlike the classically described response of the pulmonary vasculature to hypoxia, the response of IPAVA to hypoxia in the intact organism may not be mediated entirely by oxygen.

### What role do catecholamines play in recruiting IPAVA?

We were inspired to consider that IPAVA might be adrenergically regulated by (1) the observed epinephrine-induced recruitment of IPAVA in healthy humans (Laurie et al., 2012; Elliott et al., 2014), (2) our clinical observation of IPAVA in a young asthmatic patient treated with high doses of β_2_-adrengergic agonists (inhaled albuterol plus i.v terbutaline; Bates et al., 2014b), and (3) the fact that increased sympathetic tone is a common feature to both high intensity exercise and hypoxia. Isoproterenol causes vasodilation by binding the β_2_-adrengergic receptor, thereby increasing intracellular cAMP and relaxing vascular smooth muscle. Little is known about the three dimensional distribution of β_2_-adrengergic receptors in the pulmonary vasculature, but mapping their location may offer some clue as to the location of IPAVA pathways.

Interestingly Laurie et al. found using saline contrast echocardiography that propranolol did not qualitatively reduce IPAVA recruitment in hypoxic humans. These studies used a qualitative scoring mechanism that may not be sufficiently sensitive to quantify changes in IPAVA recruitment. Using our quantitative microsphere method, we found that propranolol did not completely eliminate hypoxia-induced IPAVA recruitment, but substantially reduced it. Therefore, while adrenergic blockade can limit blood flow through IPAVA, IPAVA recruitment by hypoxia was not completely prevented by propranolol. It is possible that propranolol does not completely eliminate IPAVA recruitment and that the hypoxic vasoconstriction downstream of these pathways redirects flow through the remaining patent IPAVA pathways. Alternatively, these data point to the existence of additional co-factors that may be required for complete IPAVA recruitment by hypoxia. There may be additional vasoactive neural or humoral factors that are induced by hypoxia that regulate the recruitment of IPAVA that are not blocked by propranolol.

Importantly, this work is the first to demonstrate quantitatively, using microspheres of a discreet diameter, that hyperoxia prevents blood flow through IPAVA. A potential interpretation of our *in-vivo* data is that the 15 μm microspheres pass through distended capillaries with isoproterenol, and not IPAVA. However, the fact that hyperoxia decreases microsphere passage with isoproterenol infusion suggests that this is not the case. Furthermore, hyperoxia completely antagonizes IPAVA recruitment by isoproterenol. This is in contrast to propranolol, which only partially blocked the impact of hypoxia. This suggests that hyperoxia is working through an independent mechanism.

The structure and anatomic location of these pathways remains a topic of debate and defining them morphometrically is an important next step. Weibel carefully and systemically characterized the anatomy of the pulmonary vasculature by evaluating serial histological sections of the human lung and noted no evidence of direct arteriovenous connections (Weibel, 1963). In contrast, Tobin studied the pulmonary vascular tree in lungs infused with casting material and found glomerular-like structures early in the vascular tree with direct arteriovenous connections (Tobin and Zariquiey, 1950; Tobin, 1966). He suggested that these structures are not visible in traditional histological sections because they have little elastic tissue and collapse during sectioning. It certainly is possible that, IPAVA are simply passive structures and that flow through them is determined by the tone of the vasculature surrounding them. That is to say, in our study, isoproterenol and propranolol may be changing the tone of the pulmonary arteries distal to these pathways, thereby redirecting blood flow through the lower resistance IPAVA. In support of this, we recently found that the transpulmonary passage of ^99m^Tc-albumin macroaggregate particles is higher in conditions when the pulmonary vascular resistance is higher (Bates et al., 2014a). In contrast, simply increasing pulmonary artery pressure and flow in our isolated lung preparation was not sufficient to recruit IPAVA. Furthermore, hyperoxia closes IPAVA even when the cardiac output is elevated by catecholamine infusion and exercise. This supports the theory that, even if the distal pulmonary vascular resistance does influence the degree to which IPAVA are perfused, they are not simply distended or recruited. We doubled the perfusate flow in our isolated lung prep, and this did not translate to an increase in IPAVA recruitment. We propose that intrapulmonary arteriovenous anastomoses may be biochemically recruited with their perfusion determined by the downstream vascular resistance.

Recall that IPAVA are (1) recruited by hypoxia and catecholamines, (2) closed by hyperoxia, and (3) a large component of the hypoxia response is blocked by propranolol. We cannot help but be struck by how similar these responses are to those of systemic blood vessels, leading us to speculate that IPAVA may, in fact, be systemic-like vessels. Since we first compared the recruitment of IPAVA by hypoxia in healthy, intact animals and isolated lungs, Galambos et al. have published several important morphometric studies of arteriovenous connections in human lungs from pediatric patients with lung disease, including alveolar capillary dysplasia, pulmonary hypertension, and bronchopulmonary dysplasia (Galambos et al., 2013, 2014, 2015; Acker et al., 2015; Ali et al., 2015). This group found that the right-to-left passage of mixed venous blood may occur in pediatric patients with fatal hypoxemia via anastomoses between the pulmonary arterial and bronchial circulations. The substantial communications between the pulmonary and bronchial circulations are well-documented and pulmonary artery-to-bronchial artery and bronchial artery-to-bronchial vein anastomoses having been observed in healthy lungs (Aramendia et al., 1962; Hyman et al., 1975; Charan et al., 1986; Thapar et al., 1991; Hasegawa et al., 1999). Furthermore, bronchial arteries are systemic in nature, being dilated by hypoxia, and catecholamines and constricted by hyperoxia. It is possible that the IPAVA we have been interested in may be bronchopulmonary in nature. More work is needed to understand the hemodynamic interactions between these two circulations.

### What are the limitations of this study and potential differences between this study and others?

This is the first time that we have been able to significantly increase the transpulmonary passage of large microspheres across the isolated, perfused lung, although it only increased to a peak of 0.8% in our isolated lung prep. Microsphere passage is typically trivial in the isolated lung and these data represent a 10–400 fold increase in microsphere passage. We acknowledge that this is not as large as the increase in the intact animal and it is worthwhile to speculate about the differences between our *in situ* and *in vivo* studies that may have led to this difference. The decreased IPAVA perfusion in the isolated lung may have been a result of the larger particles used in this experiment. We used 50 μm microspheres in our isolated lung studies in order to be consistent with our previous isolated lung studies (Lovering et al., 2007; Stickland et al., 2007; Bates et al., 2012) and increase our confidence that our findings are not simply the result of capillary distension. It requires >100 mmHg perfusion pressure to distend a pulmonary capillary from 6 to 50 μm (Linehan et al., 1992; Bates et al., 2012). We did not use 50 μm particles in our intact animal studies because their injection frequently results in hemodynamic instability. We have shown previously that the injection of 15 μm particles does not substantially impact pulmonary hemodynamics. In our intact animal model, 15 μm microsphere passage was >50 μm microsphere passage and the lower IPAVA flow in our isolated lungs may be because there are fewer IPAVA pathways that are >15 μm. We used 15 μm microspheres to quantify IPAVA flow in our intact studies because we they do not traverse the normoxic lung (here and Eldridge et al., 2004). Future studies may consider combining isolated lung physiology studies with histology to better understand the size distribution of IPAVA pathways.

Secondly, it is possible that there is lower IPAVA recruitment in the isolated lung because of differences in the experimental conditions between the isolated lung and intact animal. We previously described additional limitations of our isolated lung technique and believe they are equally applicable in this study (5). Total pulmonary flow is less in the isolated lung than the intact rat and the lung is positive pressure ventilated. Our isolated lung preparation is also perfused at room temperature with a non-blood perfusate that is in equilibrium with room air, giving it a non-physiological PvO_2_ and PvCO_2_. It is possible that this contributes to sub-maximal IPAVA recruitment in the isolated lung prep.

A final limitation of this study is that, given our technical limitations, we were not able to simultaneously measure cardiac output and inject microspheres into the left ventricle in our intact studies. This motivated us to perform experiments in our isolated lung model where we can control pressure and flow.

## Conclusions

Intrapulmonary arteriovenous anastomoses are recruited in the intact rat by β-adrenergic agonists, while hyperoxia antagonizes IPAVA recruitment. Furthermore, IPAVA recruitment by hypoxia can be minimized with β-adrenergic blockade, suggesting a role for these mediators in the recruitment of IPAVA by hypoxia. The fact that β-adrenergic agonists can recruit IPAVA in isolated lungs, without increased pulmonary flow, suggests they are independent mediators of IPAVA recruitment. While increased cardiac output is not required for the IPAVA recruitment, further studies aimed at evaluating the influence of biochemical mediators and hemodynamics on IPAVA perfusion are needed.

## Author contributions

Study design—MB, JJ, ME; Data collection and analysis—MB, JJ, ME; Manuscript and figure preparation—MB, JJ, ME; Approval of final manuscript—MB, JJ, ME.

## Funding

This work was supported by funding from the National Institutes of Health (5R01HL086897 and 5T32HL007654) and the University of Iowa Department of Health and Human Physiology.

### Conflict of interest statement

The authors declare that the research was conducted in the absence of any commercial or financial relationships that could be construed as a potential conflict of interest.

## Abbreviations

IPAVA: intrapulmonary arteriovenous anastomoses
PvO_2_: venous partial pressure of oxygen
PvCO_2_: venous partial pressure of carbon dioxide.
